# Significance of J waves in unexplained ventricular fibrillation among elderly populations with various comorbidities

**DOI:** 10.1016/j.hroo.2024.05.004

**Published:** 2024-05-16

**Authors:** Naoya Kataoka, Teruhiko Imamura, Keisuke Uchida, Takahisa Koi, Koichiro Kinugawa

**Affiliations:** Second Department of Internal Medicine, University of Toyama, Toyama, Japan

**Keywords:** Cardiopulmonary arrest, Ventricular fibrillation, J-wave, Heart failure, Electrocardiogram

**Key findings**

▪ The significance of J waves in unexplained cardiopulmonary arrest among elderly populations with numerous comorbidities has not been firmly established.
▪ This study emphasizes the association between J waves and ventricular fibrillation as the initially documented rhythm.
▪ J waves may suggest arrhythmogenic substrates that sustain ventricular fibrillation, irrespective of the presence of structural diseases or age.

The presence of J waves in surface electrocardiograms has been identified as a marker associated with idiopathic ventricular fibrillation (VF) in young to middle-aged healthy individuals without any underlying diseases.^1^ Moreover, recent invasive electrophysiological studies have demonstrated that the etiology of the J-wave is attributed to structural abnormalities and is involved in the maintenance of VF, instead of the occurrence of VF.^2^^,^^3^ These findings have prompted the hypothesis that the presence of J waves may extend beyond “idiopathic” VF, correlating with the terminal rhythm at the cardiac arrest event, even in cases with underlying comorbidities commonly encountered in the elderly cohort. This study focused on patients experiencing unexplained sudden cardiopulmonary arrest, by examining the presence of J waves in precardiac event electrocardiograms and investigating their correlation with the initially documented rhythm.

Consecutive patients who experienced unexplained cardiopulmonary arrest from 2014 to 2024 in our institution were retrospectively screened. The medical history was obtained by the attending physician and the primary nurse. Patients meeting the following criteria were included: (1) between 18 and 100 years of age at the time of cardiopulmonary arrest, (2) in the chronic phase of stable underlying diseases, and (3) experiencing unexplained cardiac arrest with no directly attributable to any deterioration in any of their underlying diseases. The exclusion criteria were defined as follows: (1) absence of any electrocardiograms before cardiac events; (2) presence of abnormal Q waves, ST-T abnormalities, or complete bundle branch blocks; (3) presence of acute illnesses including acute coronary syndrome, stroke, infections, or hypovolemic shock due to massive bleeding, which was determined utilizing a comprehensive workup, particularly including electrocardiography, transthoracic echocardiography, coronary angiography, or whole-body computed tomography scan including the head; (4) terminal stage of cancers; (5) trauma; or (6) suffocation. Cases that were documented to have sustained monomorphic ventricular tachycardia as the initial rhythm at cardiopulmonary arrest were also excluded. The electrocardiograms were evaluated blindly by 3 arrhythmia specialists (N.K., K.U., and T.K.). In cases of disagreement among the 3, particularly regarding parameters during atrial fibrillation, the interpretation agreed upon by 2 of them was adopted. This study received approval from the Ethics Committee of the University of Toyama, and consent from patients was obtained using an opt-out approach.

**Figure 1:**
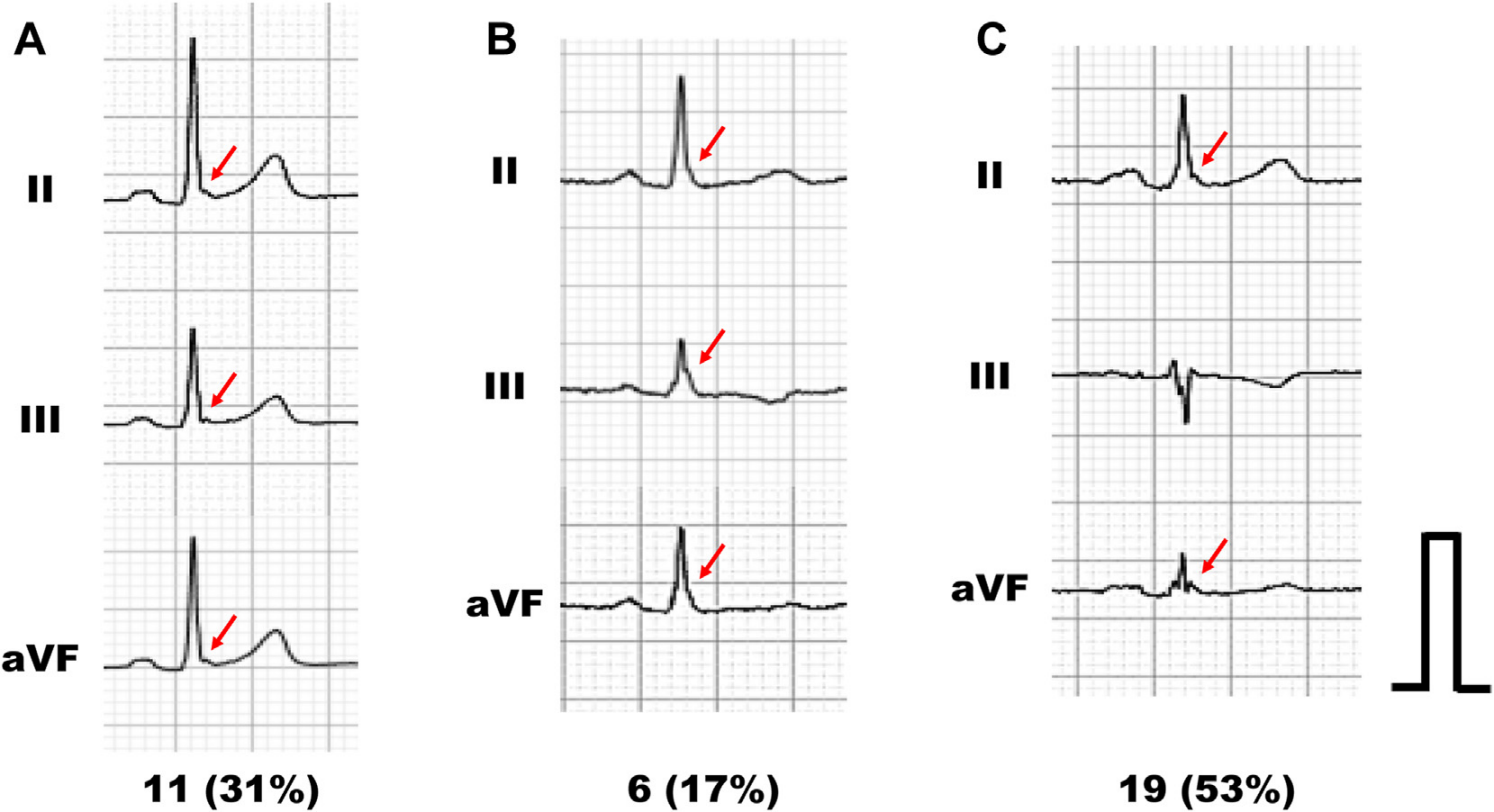
Types of J waves. A: Early repolarization with J-wave. B: Slurred QRS downstroke without ST-segment elevation. C: J-wave without ST-segment elevation. The red arrows indicate J waves.

In the results, a total of 77 patients, with a median age of 70 years at the onset of cardiopulmonary arrest, were evaluated. Baseline characteristics are depicted in Table 1. The evaluated electrocardiograms were recorded at a median of 19 (interquartile range 5.5–60.5) months prior to the unexplained cardiopulmonary arrest events. This cohort comprised 68 subjects with comorbidities, while the remaining 9 (12%) had no underlying diseases. Among them, 42 (55%) subjects had underlying heart diseases, with 17% of them having an ischemic etiology. The majority exhibited electrocardiogram parameters within the normal range; however, J waves, defined according to currently accepted definitions, were observed in 36 (47%) of the subjects in the inferior, lateral, and right precordial leads.^4^ The median maximum amplitude of the J-wave was 0.15 mV. The incidences of each type of J-wave and representative forms are displayed in Figure 1. The most frequently observed form was J-wave without ST-segment elevation (53%), characterized by a notched end of the QRS waveform. The least observed was slurred QRS downstroke without ST-segment elevation (17%). No other significant electrocardiographic abnormalities were detected.

**Table 1:** Baseline characteristics

Patient characteristics	
Age at onset of cardiopulmonary arrest, y	70 (60–79)
Ventricular fibrillation	41 (53)
Male	56 (73)
Body mass index, kg/m^2^	22.5 (19.8–24.8)
Underlying comorbidities	68 (88)
Underlying heart diseases	42 (55)
Ischemic heart disease	13 (17)
Cancers	8 (10)
Out-of-hospital onset	44 (57)
Bystander cardiopulmonary resuscitation	51 (66)
Successful return of spontaneous circulation	36 (47)
Use of extracorporeal membrane oxygenation	4 (5)
Electrocardiogram recorded prior to the onset	
Age at the time of electrocardiogram recording, years old	64 (59–74)
Atrial fibrillation	11 (14)
Heart rate, beats/min	73 (64–86)
PR interval, ms	170 (149–192)
QRS width, ms	103 (96–117)
Corrected QT interval, ms	439 (415–458)
J-wave	36 (47)
Maximum amplitude of J-wave, mV	0.15 (0.12–0.18)
Inferior leads	30 (83)
Lateral leads	10 (28)
Right precordial leads	2 (6)

Values are median (interquartile range) or n (%).

The electrocardiographic markers correlated with VF were investigated (Table 2). The incidence of VF was significantly associated with age, male sex, and underlying heart diseases (*P* < .05 for all). Adjusting for these statistically significant baseline characteristics, slow heart rate and the presence of J waves were independently correlated with the onset of VF. The electrophysiological features related to J waves were also investigated (Table 3). The age at the time of the recorded electrocardiogram was not associated with the presence of J waves. A low heart rate and a short QT interval corrected by Bazzett's formula were found to be associated with the presence of J waves. In the results of the multivariable analysis, a short QT interval was identified as an independently correlated marker with J waves with an adjusted odds ratio of 0.83 (95% confidence interval 0.71-0.97; *P* = .015).

**Table 2:** Electrocardiographic parameters associated with the onset of ventricular fibrillation

	Univariable			Adjusted for age, sex, and comorbidities		
	Odds ratio	95% CI	*P* value	Odds ratio	95% CI	*P* value
Patient characteristics						
Age at recorded electrocardiogram, per 10-y increase	0.68	0.47–0.97	.024			
Male	3.09	1.08–8.87	.036			
Body mass index, per 5-kg/m^2^ increase	0.76	0.45–1.29	.298			
Underlying comorbidities	0.29	0.06–1.48	.135			
Underlying heart diseases	5.45	2.05–14.51	.001			
Ischemic heart disease	6.23	1.28–30.40	.024			
Cancers	0.26	0.05–1.36	.256			
Electrocardiogram						
Atrial fibrillation	0.69	0.19–2.50	.577			
Heart rate, per 10-beats/min increase	0.62	0.45–0.86	.002	0.57	0.39–0.85	.002
PR interval, per 10-ms increase	1.10	0.94–1.27	.199			
QRS width, per 10-ms increase	1.24	0.99–1.56	.042	1.08	0.84–1.39	.524
Corrected QT interval, per 10-ms increase	1.00	0.87–1.15	.998			
The presence of J waves	13.63	4.47–41.63	<.001	18.66	4.52–77.08	<.001

CI = confidence interval.

**Table 3:** Correlation between electrophysiological parameters and J waves

	Univariable			Multivariable		
	Odds ratio	95% CI	*P* value	Odds ratio	95% CI	*P* value
Patient characteristics						
Age at recorded electrocardiogram, 10-y increase	0.75	0.53–1.04	.075			
Male	1.62	0.58–4.53	.353			
Body mass index, 5-kg/m^2^ increase	1.00	0.60–1.66	1.000			
Underlying comorbidities						
Underlying heart diseases	1.65	0.67–4.09	.280			
Ischemic heart disease	3.08	0.86–11.07	.084			
Cancers	0.65	0.14–2.95	.582			
Electrocardiogram						
Atrial fibrillation	1.44	0.40–5.19	.577			
Heart rate, 10 beats/min increase	0.75	0.55–1.00	.048	0.76	0.56–1.03	0.072
PR interval, 10-ms increase	1.11	0.96–1.28	.139			
QRS width, 10-ms increase	0.99	0.82–1.20	.909			
Corrected QT interval, 10-ms increase	0.83	0.71–0.96	.010	0.83	0.71–0.97	.015

CI = confidence interval.

In summary, the present retrospective cohort study has revealed the following findings: (1) the presence of J waves and also lower heart rate was associated with VF occurrence, even in elderly cases with underlying comorbidities; and (2) J waves were more likely to occur together with shorter QT interval.

A previous experimental research indicated that healthy cardiac tissue is unable to sustain VF due to the collision between the wavefront and ventricular refractory periods, resulting in its spontaneous termination.^4^ According to the cited study, the VF wavelength was determined by the product of action potential duration and conduction velocity.^4^ The etiology of J waves has generally been attributed to pronounced notching in the epicardial action potential caused by Ito overcurrent, accompanied by a simultaneous reduction in action potential duration on the epicardium. Indeed, in the present study, the presence of J waves was associated with a short QT interval, which is determined by the action potential duration (Table 3).^5^ These findings suggested that a short action potential duration plays a crucial role in maintaining VF. Subjects without J waves might exhibit normal action potential duration and conduction velocity, indicating the spontaneous termination of the reentry of VF waveform, resulting in pulseless electrical activity or asystole as the initial documented rhythm in the setting with cardiopulmonary arrest.

Furthermore, not only J waves, but also lower heart rate were associated with the occurrence of VF (Table 2). A reduced heart rate has been acknowledged as a factor contributing to the presence of J waves.^6^ However, in this cohort, no statistically significant association was observed between heart rate and J waves. Vagal activity, established as a contributor to a decreased heart rate, may play a role in enhancing J waves, potentially leading to the onset of VF.^7^

The limitations were pointed out as follows. First, pathological examinations such as genetic testing, histology, and imaging studies directly suggesting the relationship between J waves and histological abnormalities were not conducted. Especially, the decrease in left ventricular ejection fraction may be strongly associated with the VF occurrence.^8^ However, in this study, echocardiographic evaluations before VF onset were not performed in many cases, and therefore could not be assessed. The possibility of including idiopathic VF cannot be ruled out, but it is considered to be low, with only 9 subjects. Second, the reasons why the prevalence of J waves in this cohort was high compared with previous studies focused on younger individuals with idiopathic VF were not fully understood.^9^ However, an association between J waves and structural heart diseases has also been proposed.^3^ Therefore, it is plausible that the higher prevalence of J waves in this cohort, which included a substantial number of cases with comorbidities including structural heart diseases, may contribute to this disparity. While the inclusion of patients with underlying heart diseases represented a significant limitation, it appeared justifiable to integrate chronic heart conditions for the assessment of electrocardiographic features correlate with VF onset as a terminal event in human mortality. Finally, patient characteristics, including age, should be evaluated as contributors to VF occurrence; however, the small number of VF cases limited the statistical power for multivariable analyses.

In conclusion, the pre-existing J waves were associated with VF in cases of unexplained cardiopulmonary arrest, even in elderly individuals with underlying comorbidities. The result, indicating that the presence of J waves was associated with a short QT interval, suggests the mechanisms of VF as the initially documented rhythm.
